# A Care-Level-Stratified Algorithm for Neuromodulation in Persistent Spinal Pain Syndrome Type 2: Critical Appraisal of Contemporary Evidence Adapted to Latin American Health Systems

**DOI:** 10.7759/cureus.117149

**Published:** 2026-09-29

**Authors:** Juan J Valero Quintero, Héctor Aceituno, Francisco Rico-Fernández, José Vásconez-Fabre, Juan Lopéz-Urdaneta, Eugenio Cerezo-Prado, José Villamediana-Rodríguez, Jennifer Vela-Poveda, Luisana Maldonado-De Santiago, Anthuan Hazkour

**Affiliations:** 1 Neurosurgery, Central University of Venezuela, Caracas, VEN; 2 Neurosurgery, Hospital San Juan de Dios de Curicó, Curicó, CHL; 3 Neurosurgery, Clínica Miguel Claro, Santiago, CHL; 4 General Medicine, Cesfam Oriente, San Fernando, CHL; 5 General Medicine, Servicio de Alta Resolutividad (SAR) Bombero Garrido, Curicó, CHL

**Keywords:** dorsal root ganglion stimulation, failed back surgery syndrome, health equity, latin america, low back pain, neuromodulation, persistent spinal pain syndrome, spinal cord stimulation, treatment algorithm

## Abstract

Persistent spinal pain syndrome type 2 (PSPS-T2), the mechanism-based successor to failed back surgery syndrome (FBSS) and a recognized International Classification of Diseases 11th Revision (ICD-11) entity, affects 10%-40% of patients after spinal surgery and is among the most refractory conditions in pain medicine. The past decade has produced a technological expansion in neuromodulation, with next-generation modalities (10-kHz high-frequency stimulation (HF10; Nevro Corp., Redwood City, CA), burst stimulation (Abbott, Plano, TX), differential target multiplexed (DTM) stimulation (Medtronic, Minneapolis, MN), evoked compound action potential-controlled closed-loop stimulation (ECAP-CL; Saluda Medical, Artarmon, Australia), dorsal root ganglion stimulation (DRG-S; Abbott, Plano, TX), and restorative neurostimulation (ReActiv8; Mainstay Medical, Dublin, Ireland)) each supported by randomized controlled trials (RCTs). We conducted a narrative review designed to meet the Scale for the Assessment of Narrative Review Articles (SANRA) quality criteria, searching five databases (January 2020 to April 2026) to critically appraise this evidence, including negative results and methodological controversies, and to propose a mechanism-directed treatment algorithm stratified by level of care and adapted to the heterogeneous resource landscape of Latin American health systems. Pivotal trials report responder rates of approximately 74%-78% at 12-36 months, with superiority over conventional stimulation; however, these estimates derive from trials that differ in population, comparator, and follow-up, and several enrolled populations only partially overlapping with PSPS-T2 (e.g., complex regional pain syndrome or non-surgical mechanical low back pain). The single independent, placebo-controlled crossover trial (of burst stimulation) found no benefit on its primary disability outcome, underscoring the field's near-total reliance on manufacturer-sponsored studies. On this basis, we propose a three-tier algorithm (primary, secondary, and tertiary care) that rests on Oxford Centre for Evidence-Based Medicine (OCEBM) level 1-2 evidence for individual modality efficacy and on expert consensus (OCEBM level 5) for the stratification itself. The algorithm is intended as a pragmatic, resource-sensitive decision-support scaffold requiring local adaptation and prospective regional validation. Equitable access to neuromodulation across Latin America remains a first-order ethical and health-policy priority.

## Introduction and background

Persistent spinal pain syndrome type 2 (PSPS-T2), the mechanism-centered successor to the term failed back surgery syndrome (FBSS), formalized by Christelis et al. [1] in 2021 and recognized in the International Classification of Diseases 11th Revision (ICD-11) (MG30.21), affects between 10% and 40% of patients following spinal surgery and represents one of the most therapeutically challenging conditions in contemporary neurosurgery and pain medicine [2,3]. Recent consensus work has extended this framework into mechanism-based clinical recommendations [4]. Its pathophysiology integrates peripheral and central sensitization, dorsal-horn neuroinflammation, perineural fibrosis, and maladaptive cortical reorganization, mechanisms that standard analgesic pharmacotherapy and repeat surgery address incompletely at best [5,6].

Since the PROCESS trial established spinal cord stimulation (SCS) superiority over conventional medical management for FBSS in 2007 [7], the field has undergone rapid technological change [8]. Next-generation modalities (10-kHz high-frequency stimulation (HF10; Nevro Corp., Redwood City, CA), burst stimulation (Abbott, Plano, TX), differential target multiplexed (DTM) stimulation (Medtronic, Minneapolis, MN), evoked compound action potential-controlled closed-loop stimulation (ECAP-CL; Saluda Medical, Artarmon, Australia), dorsal root ganglion stimulation (DRG-S; Abbott, Plano, TX), and restorative neurostimulation (ReActiv8; Mainstay Medical, Dublin, Ireland)) have each emerged with randomized controlled trial (RCT) evidence [9-14].

For Latin America, access to these technologies is profoundly heterogeneous. Whereas tertiary neurosurgical centers in Argentina, Brazil, Chile, Colombia, and Mexico offer most contemporary modalities, the majority of the region's population is cared for in primary and secondary facilities where basic neuromodulation may be unavailable [15]. This reality demands a treatment algorithm stratified not merely by clinical phenotype but by healthcare resource level, an explicit design priority of this review. Throughout, Latin America is defined pragmatically as the Spanish-speaking countries and territories of the region together with Brazil (20 jurisdictions in total).

This review pursues two objectives: first, to critically appraise the 2020-2026 evidence for contemporary neuromodulation modalities in PSPS-T2, including negative results, indirect evidence, and industry-sponsorship bias; and second, to propose a mechanism-directed treatment algorithm stratified by level of care and calibrated to the heterogeneous resource landscape of Latin American health systems.

## Review

Methodology

This is a narrative (non-systematic) review, designed to satisfy the quality domains of the Scale for the Assessment of Narrative Review Articles (SANRA) [16]. It is not a systematic review: it does not include a Preferred Reporting Items for Systematic Reviews and Meta-Analyses (PRISMA) flow diagram, duplicate independent screening, or quantitative synthesis, and it is therefore subject to selection bias, which we mitigated by pre-specifying the modalities of interest and by including negative and non-industry evidence.

We searched five databases (PubMed/MEDLINE, Cochrane CENTRAL, Scopus, Web of Science, and EMBASE) for the primary period January 2020 to April 2026, with seminal earlier trials retained for context. The last search was run in April 2026. Search terms combined controlled vocabulary and free text for the population and interventions, for example, ("persistent spinal pain syndrome" OR "failed back surgery syndrome") AND ("spinal cord stimulation" OR "burst" OR "high frequency" OR "closed loop" OR "dorsal root ganglion" OR "restorative neurostimulation"). Inclusion was restricted to RCTs, systematic reviews, network meta-analyses, and clinical practice guidelines published in English, Spanish, or Portuguese; narrative commentaries, preclinical studies, and single-arm case series were excluded from the evidence table.

Each pivotal study was graded with the Oxford Centre for Evidence-Based Medicine (OCEBM) 2011 levels of evidence [17] and independently appraised across the five domains of the Cochrane risk-of-bias 2 (RoB 2) tool [18]. Because RoB 2 does not include funding source as a domain, industry sponsorship (pervasive in neuromodulation trials and a documented source of optimism bias [19,20]) is reported separately and discussed below. The proposed algorithm is labeled throughout as an expert-consensus proposal (OCEBM level 5) for its stratification component, built upon OCEBM level 1-2 evidence for individual modality efficacy, and requiring prospective regional validation.

Evidence for contemporary neuromodulation modalities

Table 1 summarizes the pivotal evidence for each modality, including the population actually studied, the comparator, the primary outcome and follow-up, the funding source, and the OCEBM level.

**Table 1:** Pivotal evidence for contemporary neuromodulation modalities in PSPS-T2 CLBP: chronic low back pain, CMM: conventional medical management, CRPS: complex regional pain syndrome, DRG-S: dorsal root ganglion stimulation, DTM: differential target multiplexed, ECAP-CL: evoked compound action potential-controlled closed-loop, FBSS: failed back surgery syndrome, HF10: 10-kHz high-frequency, ODI: Oswestry Disability Index, OCEBM: Oxford Centre for Evidence-Based Medicine, PSPS-T2: persistent spinal pain syndrome type 2, RCT: randomized controlled trial, SCS: spinal cord stimulation, VAS: visual analog scale Populations that differ from PSPS-T2 are discussed under the indirectness of evidence.

Modality (device; manufacturer)	Pivotal trial	Number	Population studied	Design/comparator	Primary outcome; follow-up	Key result	Sponsor	OCEBM
Conventional tonic SCS	PROCESS [7]	100	FBSS (post-surgical)	RCT; SCS + CMM versus CMM	≥50% leg-pain relief; 6 months	48% versus 9% responders	Public/academic	2
HF10/10-kHz (Senza; Nevro Corp.)	SENZA-RCT [9,21]	198	Chronic back + leg pain (including post-surgical)	RCT; HF10 versus tonic SCS	Back-pain responder; 3-24 months	76.5% versus 49.3% at 24 months	Nevro Corp.	2
Burst (Proclaim; Abbott)	SUNBURST [10]	100	Chronic trunk/limb pain (including FBSS)	RCT crossover; burst versus tonic SCS	Overall pain (VAS); 24 weeks	Superiority; patient preference for burst	Abbott	2
Burst (independent replication)	Hara et al. [22]	50	Chronic radicular pain after lumbar surgery (PSPS-T2)	RCT, placebo-controlled crossover (4 × 3 months)	ODI change; primary + 6-month follow-up	No superiority versus placebo	Investigator-initiated (non-industry)	2
ECAP-CL (Evoke; Saluda Medical)	Evoke [11,23]	134	Chronic back + leg pain (including post-surgical)	RCT, double-blind; closed- versus open-loop	≥50% responder; 12-36 months	77.6% versus 49.3% at 36 months	Saluda Medical	2
DRG-S (Proclaim DRG; Abbott)	ACCURATE [12]	152	CRPS/causalgia (not PSPS-T2)	RCT; DRG-S versus tonic SCS	Treatment success; 3-12 months	74.2% versus 53.0% at 12 months	Abbott (Spinal Modulation)	2
Restorative neurostim. (ReActiv8; Mainstay)	ReActiv8-B [14]	204	Mechanical CLBP with multifidus dysfunction, non-surgical (not PSPS-T2)	RCT, sham-controlled	Pain (VAS) responder; primary	Primary endpoint not met: 57.1% versus 46.6% responders at 120 days (p = 0.138)	Mainstay	2
DTM (Intellis; Medtronic)	Fishman et al. [13]	128	Chronic back + leg pain	RCT, open-label; DTM versus traditional SCS	Low-back-pain responder; 3 months (12-month results reported)	Superiority in low-back-pain responder rate at 3 and 12 months	Medtronic (Stimgenics)	2

A recurring theme is that responder rates cluster at roughly 73%-77% at 12-36 months, but the underlying trials differ substantially in population and comparator, so these figures should not be pooled or read as directly interchangeable.

HF10 stimulation

HF10 (10 kHz, subthreshold, paresthesia-free) demonstrated superiority over conventional tonic SCS in the SENZA-RCT, with a back-pain responder rate of 76.5% versus 49.3% [9]; the 24-month data confirmed durability of this advantage [21]. Both publications were sponsored by the manufacturer (Nevro Corp.), a limitation that should temper interpretation.

Burst stimulation and the Hara trial

The SUNBURST trial showed burst superiority over tonic SCS at the primary endpoint, with a patient preference for burst [10]. However, the placebo-controlled crossover trial by Hara et al., the only rigorously blinded, placebo-controlled trial in the field and, notably, an investigator-initiated study without device-manufacturer funding, randomized 50 patients with chronic radicular pain after lumbar spine surgery and found no superiority of burst stimulation over placebo on the primary Oswestry Disability Index outcome [22]. This is directly relevant because the enrolled population overlaps substantially with PSPS-T2. The contrast suggests that superiority over conventional SCS may partly reflect a placebo-expectation differential rather than a specific effect of the burst waveform; as an OCEBM level 2 study conducted independently, it cannot be dismissed.

Evoked compound action potential-controlled closed-loop stimulation

The Evoke trial demonstrated ECAP-CL superiority over open-loop SCS in a double-blind design, with 36-month follow-up showing sustained advantages (77.6% versus 49.3% responder rate; between-group difference: 28.4%, p < 0.001) [11,23]. The device continuously adjusts output to maintain a target evoked compound action potential, theoretically providing more consistent dorsal-horn activation. Industry sponsorship (Saluda Medical) was present.

Dorsal root ganglion stimulation

The ACCURATE trial demonstrated DRG-S superiority over conventional SCS at 12 months for complex regional pain syndrome and causalgia (74.2% versus 53.0% treatment success) [12]. DRG-S enables precise dermatomal coverage, potentially useful for focal lower-limb neuropathic pain in PSPS-T2; best-practice recommendations have been published [24]. Because the pivotal population was CRPS/causalgia rather than PSPS-T2, application to PSPS-T2 constitutes indirect evidence (see below).

Restorative neurostimulation

The ReActiv8-B trial evaluated restorative neurostimulation against sham for mechanical chronic low back pain with multifidus dysfunction but did not meet its primary endpoint: the between-group difference in responder rate at 120 days was not statistically significant (57.1% versus 46.6%; p = 0.138) [14]. Reported efficacy therefore rests on secondary and longer-term open-label analyses rather than on the primary sham-controlled comparison. The device stimulates the medial branch of the dorsal ramus to elicit therapeutic multifidus contraction, a mechanism distinct from pain modulation that targets muscular deconditioning. Its studied population comprised non-surgical patients with documented multifidus dysfunction and no predominant radicular pain, which again differs from classic PSPS-T2. (We note that the pivotal sham-controlled study is the ReActiv8-B trial; a separate trial against optimal medical management, RESTORE, should not be conflated with it.)

Differential target multiplexed stimulation

The Fishman et al. open-label RCT randomized 128 subjects and showed superiority of DTM over traditional SCS in the low-back-pain responder rate at the 3-month primary endpoint, sustained through 12 months [13]. The open-label design (patients and treating physicians were not blinded) is a notable limitation, and the trial was manufacturer-supported.

Pharmacological and non-interventional management

Neuropathic pain, the predominant mechanism in most patients with PSPS-T2, is best addressed pharmacologically per the Finnerup et al. systematic review [25]: first-line agents are gabapentinoids and serotonin-norepinephrine reuptake inhibitors, with tricyclic antidepressants as second-line owing to tolerability concerns. The 2022 CDC guideline discourages prolonged opioid therapy for chronic non-cancer low back pain [26]. Cochrane evidence supports motor-control exercise [27] and psychological therapies including cognitive behavioral therapy [28] within multimodal management. A recent meta-analysis characterizes the limited benefit of conventional medical management alone in this refractory population [29], reinforcing the rationale for structured, mechanism-based escalation.

Proposed care-level-stratified algorithm for Latin American settings

The algorithm (Table 2) is an expert-consensus proposal (OCEBM level 5) for its stratification, built on OCEBM level 1-2 evidence for individual modalities.

**Table 2:** Proposed care-level-stratified algorithm for neuromodulation in PSPS-T2, for local adaptation across Latin American health systems CSI: Central Sensitization Inventory, DN4: Douleur Neuropathique 4, DRG-S: dorsal root ganglion stimulation, DTM: differential target multiplexed, ECAP-CL: evoked compound action potential-controlled closed-loop, HF10: 10-kHz high-frequency, NACC: Neurostimulation Appropriateness Consensus Committee, OCEBM: Oxford Centre for Evidence-Based Medicine, ODI: Oswestry Disability Index, PCS: Pain Catastrophizing Scale, PSPS-T2: persistent spinal pain syndrome type 2, SNRI: serotonin-norepinephrine reuptake inhibitor, TSK: Tampa Scale for Kinesiophobia The named psychometric instruments are cited to their validation literature; some (e.g., the Pain Catastrophizing Scale) require licensing for clinical or research use.

Level of care	Typical setting	Core clinical actions	Evidence basis
Level 1: primary care	All settings, including resource-limited	Mechanism-based phenotyping with validated instruments (DN4 [30], painDETECT [31], CSI [32], ODI [33], PCS [34], TSK [35]); first-line pharmacotherapy with gabapentinoids and SNRIs [25]; structured active rehabilitation and motor-control exercise [27]; cognitive behavioral therapy where available [28]; explicit avoidance of prolonged opioid therapy [26]	OCEBM 1-2 (components); level 5 (sequencing)
Level 2: secondary care	Regional hospital (neurosurgery or pain medicine)	Confirmatory mechanism-based phenotyping; epidural interventions for a predominantly nociceptive component [36]; restorative neurostimulation for mechanical low back pain with multifidus dysfunction [14]; conventional SCS trial for predominantly neuropathic leg pain meeting NACC criteria [37,38]	OCEBM 2 (modalities); level 5 (stratification)
Level 3: tertiary care	Specialized neuromodulation center	Next-generation modality by phenotype: HF10 for combined back and leg pain [9,21]; ECAP-CL for closed-loop dose titration [11,23]; DRG-S for focal lower-limb neuropathic pain [12,24]; DTM for a predominant central-sensitization phenotype [13]; multidisciplinary integration as standard; industry-sponsorship bias explicitly factored into device selection	OCEBM 2 (modalities); level 5 (selection)

It is organized by three levels of care that approximate, but do not map uniformly onto, the resource landscape of the region: the same nominal tier carries very different capabilities across and within countries. We therefore present it as a scaffold to be localized by each health system rather than as a ready-to-deploy regional standard, and the following section specifies the contextual determinants and system archetypes that govern its local adaptation.

Critical appraisal, risk of bias, and limitations

We appraised each pivotal randomized trial with the Cochrane RoB 2 tool [18]; the domain-by-domain judgments are shown in Table 3.

**Table 3:** Cochrane RoB 2 assessment of the pivotal randomized trials, by domain (authors' appraisal) RoB 2 domains per Sterne et al. [18] Judgments: Low (low risk of bias), some concerns, or high (high risk of bias) Domain D4 is the dominant limitation in trials comparing paresthesia-free waveforms with paresthesia-based tonic stimulation, because the perceptible paresthesia difference precludes patient blinding for subjective outcomes; the open-label DTM trial carries the same limitation by design. Sham- or placebo-controlled blinded trials (Hara et al.; ReActiv8-B) and the double-blind closed- versus open-loop comparison (Evoke) reach lower risk. Funding source is not a RoB 2 domain and is reported separately in Table 1. DTM: differential target multiplexed, RoB 2: risk-of-bias 2

Trial	D1: Randomization process	D2: Deviations from intended interventions	D3: Missing outcome data	D4: Measurement of the outcome	D5: Selection of the reported result	Overall
PROCESS, Kumar et al. (2007) [7]	Some concerns	High	Some concerns	High	Some concerns	High
SENZA-RCT, Kapural et al. (2015) [9,21]	Low	Some concerns	Low	High	Some concerns	High
SUNBURST, Deer et al. (2018) [10]	Low	High	Some concerns	High	Some concerns	High
Hara et al. (2022) [22]	Low	Low	Low	Low	Low	Low
Evoke, Mekhail et al. (2020/2024) [11,23]	Low	Low	Some concerns	Low	Low	Some concerns
ACCURATE, Deer et al. (2017) [12]	Low	Some concerns	Some concerns	High	Some concerns	High
ReActiv8-B, Gilligan et al. (2021) [14]	Low	Some concerns	Some concerns	Some concerns	Low	Some concerns
DTM, Fishman et al. (2021) [13]	Low	High	Some concerns	High	Some concerns	High

A clear gradient emerges: the paresthesia difference between newer paresthesia-free waveforms and conventional tonic stimulation precludes patient blinding, so trials comparing the two carry a high risk of bias in outcome measurement for subjective pain and disability endpoints, and open-label designs compound this. By contrast, the sham- or placebo-controlled trials and the double-blind closed- versus open-loop comparison achieve lower risk.

This appraisal sharpens the interpretation of the efficacy data. The only trial at overall low risk of bias that was also independently funded (Hara et al.) failed to show benefit over placebo, whereas the trials at high risk of bias were uniformly positive. Beyond blinding, industry sponsorship is near-universal: every manufacturer-branded pivotal trial reviewed (SENZA-RCT, SUNBURST, Evoke, ACCURATE, ReActiv8-B, and the DTM trial) was funded by the device maker [19,20,22]. The field's reliance on manufacturer-sponsored studies for its level 1-2 evidence is a structural vulnerability that independent academic and registry research must address.

Second, much of the evidence is indirect with respect to PSPS-T2. ACCURATE enrolled complex regional pain syndrome and causalgia [12], ReActiv8-B enrolled non-surgical mechanical low back pain with multifidus dysfunction [14], and the best available indirect comparison (the Eldabe et al. network meta-analysis) addresses non-surgical refractory back pain [39]. Extrapolating these results to PSPS-T2 lowers the certainty of the corresponding recommendations, and the algorithm should be read with that downgrade in mind.

Third, no head-to-head trials compare next-generation modalities against one another in PSPS-T2; comparisons are almost all against conventional SCS. The stratification component of the algorithm therefore rests on expert consensus rather than direct comparative evidence, and its central-sensitization-to-DTM and phenotype-to-device mappings are hypotheses for validation, not established standards. Finally, as a narrative review without duplicate screening or quantitative synthesis, this work is itself subject to selection bias.

Contextual determinants and system archetypes for local adaptation

Because the three-tier structure assumes capabilities that are unevenly distributed, applying the algorithm requires an explicit overlay of contextual determinants that operate above the clinical phenotype. Four are decisive. The first is financing and benefit-package inclusion: Latin American systems are markedly segmented, mixing tax-funded public provision, social-health-insurance funds, and private insurance in different proportions, so whether a device costing tens of thousands of US dollars is reachable depends less on the existence of a tertiary center than on whether the relevant scheme covers it [15,40]. Where inclusion is absent or discretionary, access frequently shifts to individual right-to-health litigation, a widespread and inequitable pathway in Argentina, Brazil, and Colombia that favors those able to litigate [41]. The second is device registration and commercial availability: each country operates its own regulatory authority and market, so a given next-generation modality may be registered and distributed in some jurisdictions and unavailable in others, independent of clinical need. The third is workforce and follow-up infrastructure: implantation, programming, and long-term titration require trained implanters and reliable device follow-up that are concentrated in a few urban centers. The fourth is referral pathways: the algorithm presupposes functioning escalation from primary to tertiary care, which is not uniform across segmented systems [40].

The economic case must also be localized. Cost-effectiveness estimates for spinal cord stimulation were generated against high-income willingness-to-pay thresholds, whereas opportunity-cost-based thresholds for the region are substantially lower, below one gross-domestic-product-per-capita per quality-adjusted life-year, and below half of that in many upper-middle-income countries [42]. A modality that is cost-effective in a high-income system may therefore not be cost-effective at regional thresholds, and the incremental cost-effectiveness ratio should be recomputed locally before adoption.

To make local adaptation operational, Table 4 groups systems into pragmatic archetypes defined by two dominant axes (the degree of system integration and the mechanism by which high-cost neuromodulation is financed), with representative examples.

**Table 4:** System archetypes for local adaptation of the algorithm across Latin American health systems (authors' pragmatic typology) HTA: health-technology assessment, SUS: Sistema Único de Saúde Archetypes are grouped by system architecture, not by device-specific reimbursement status, which varies and changes over time; representative settings are illustrative, and intranational variation can be substantial. Classification of system integration follows the Latin American universal-health-coverage literature [15,40].

Archetype	System integration and financing	Typical access route to advanced neuromodulation	Representative settings	Implication for the algorithm
Integrated tax-funded universal	Single public payer; broad population coverage, but high-cost device inclusion is often limited or explicitly rationed	Public benefit package where the device is included; otherwise litigation	Brazil (SUS)	Tiers 1-2 broadly deliverable; tier 3 depends on explicit public inclusion
Mandatory insurance with a defined benefit plan	Contributory and subsidized insurance with an explicit, HTA-informed benefits package	Access if the device is listed in the plan; high-cost mechanisms or litigation otherwise	Colombia	Tier 3 gated by benefit-plan listing and health-technology assessment
Segmented social-security plus private	Parallel social-security funds and private insurance with a large out-of-pocket share	Varies by fund or insurer; out-of-pocket payment or litigation common	Argentina, Chile, Mexico	Tier 3 reachable in well-resourced sub-systems, not uniformly
Resource-constrained	Limited specialist infrastructure and no routine financing of high-cost devices	Advanced neuromodulation largely unavailable domestically	Several Central American countries, Bolivia, Haiti	Emphasis on tiers 1-2; tier 3 typically requires referral elsewhere

The archetype, rather than the country label, indicates which tier of the algorithm is realistically reachable and by what route. The typology is deliberately coarse and time-sensitive: benefit packages, device registrations, and case law change, and intranational heterogeneity (e.g., between metropolitan and rural regions of large federal countries) can exceed differences between countries [40].

Two contrasting cases illustrate the overlay. A patient with predominantly neuropathic leg pain at a tertiary public center in an integrated system may satisfy every clinical criterion for a next-generation modality yet reach it only if the device is publicly funded or obtained through litigation, so the rate-limiting step is financing, not phenotype. Conversely, a patient with the same phenotype in a secondary hospital of a resource-constrained system is realistically confined to tier 1-2 care (mechanism-based pharmacotherapy, rehabilitation, and, where available, epidural interventions), with tier 3 reachable only by referral to a distant or foreign center. In both cases, the phenotype identifies what is ideal; the contextual determinants identify what is possible.

For the region as a whole, equitable access to neuromodulation is therefore both a clinical and a health-policy problem: resource availability ranges from highly specialized centers in Santiago, São Paulo, Buenos Aires, and Mexico City to primary-care facilities in rural areas of several countries [15]. An algorithm that is explicit about these determinants, rather than one assuming uniform access to tertiary technology, is a practical necessity, and it points toward the regional registries, health-technology-assessment processes, and comparative studies needed to validate and localize it.

## Conclusions

Contemporary neuromodulation modalities for PSPS-T2 are supported by randomized evidence of efficacy, but that evidence rests almost entirely on manufacturer-sponsored trials, and the single independent, placebo-controlled study tempers enthusiasm for at least one widely used waveform. Much of the supporting data is also indirect, having been generated in populations that only partially overlap with PSPS-T2. These features call for cautious interpretation and for independent, comparative, and sham-controlled research.

The care-level-stratified algorithm proposed here is an expert-consensus framework that combines evidence-based modality efficacy with a resource-calibrated structure for the countries of Latin America. It is offered as a pragmatic decision-support tool rather than a definitive standard, and its stratification requires prospective regional validation. Equitable access to neuromodulation across the region remains a first-order ethical and health-policy priority, requiring sustained investment in clinical research, specialized training, and institutional infrastructure.
